# The Effect of Alcohol Consumption in Unresectable Hepatocellular Carcinoma with Transarterial Chemoembolization

**DOI:** 10.1155/2022/7062105

**Published:** 2022-12-30

**Authors:** Bo Sun, Lijie Zhang, Dongqiao Xiang, Qing Li, Yanqiao Ren, Yanyan Cao, Tao Sun, Weihua Zhang, Linxia Wu, Licheng Zhu, Lei Chen, Huangxuan Zhao, Chuansheng Zheng

**Affiliations:** ^1^Department of Radiology, Union Hospital, Tongji Medical College, Huazhong University of Science and Technology, Wuhan 430022, China; ^2^Hubei Province Key Laboratory of Molecular Imaging, Wuhan 430022, China; ^3^Department of Interventional Radiology, Union Hospital, Tongji Medical College, Huazhong University of Science and Technology, Wuhan 430022, China; ^4^Department of Interventional Radiology, The Fifth Medical Center of Chinese, PLA General Hospital, Beijing 100039, China

## Abstract

**Background:**

Alcohol consumption can increase the risk of developing hepatocellular carcinoma (HCC). However, whether continuous alcohol consumption can influence outcomes in patients with HCC who undergo transarterial chemoembolization (TACE) remains unclear. This study aimed to explore the effect of alcohol consumption in patients with unresectable HCC who underwent TACE.

**Methods:**

The data used in the study were obtained from two centers and were retrospectively reviewed between January, 2014, and December, 2021. 254 patients with TACE were included in this study. Among them, 101 patients were continuous alcohol consumers and 153 patients had alcohol abstinence. Propensity score matching (PSM) and competing risk analysis were used to reduce the selection bias.

**Results:**

The median overall survival (mOS) and median progression-free survival (mPFS) in the alcohol consumers' group were longer than those in the alcohol abstinence group, before and after PSM. Multivariate regression analysis showed that alcohol consumption increased all-cause mortality risk (HR: 1.486, 95% CI: 1.074–2.055; *P*=0.016) and tumor progression risk (HR: 1.434, 95% CI: 1.091–1.886; *P*=0.01) more than that with alcohol abstinence. In the competing risk analysis, after excluding deaths caused by other reasons, alcohol consumption increased cancer-specific mortality risk more than alcohol abstinence did before and after PSM. Adverse event analysis showed that alcohol consumption increased the risk of all grades of nausea and vomiting and grade III or IV nausea more than alcohol abstinence did after patients underwent TACE.

**Conclusion:**

Alcohol consumption may lead to a poor prognosis and increase adverse events in patients receiving TACE compared to those with alcohol abstinence.

## 1. Introduction

Primary liver cancer is the fourth most common malignant cancer and the third most lethal cancer in China [1, 2]. HCC is the main histological type of liver cancer, accounting for approximately 90% of all liver cancer cases. Alcohol-related liver disease contributes to approximately one-third of the hepatocellular carcinoma cases worldwide and is the main driver of liver carcinogenesis in the US and many European countries [3]. Hepatitis B virus (HBV) is the leading cause of HCC in China, but alcohol consumption is also a main cause of HCC [4, 5]. Studies have suggested that alcohol consumption may increase the risk of HCC and shorten survival in the population and may also lead to a poor prognosis in patients with cirrhosis [6–9]. However, the influence of alcohol on patients with HCC who receive related treatment remains unclear and needs to be clarified.

Patients with early HCC are recommended to undergo liver transplantation, liver resection, or radiofrequency ablation (RFA) because these treatments can prolong the 5-year survival [10–12]. However, most patients diagnosed with HCC are in intermediate or advanced stages. For such patients, TACE, tyrosine kinase inhibitors (TKI), or TKI combined with immunotherapy are recommended as the first-line treatments [11, 13]. TACE has been widely used in the treatment of intermediate-stage HCC because a randomized controlled trial conducted by Josep M. Llovet et al. showed that TACE could prolong the survival of patients with intermediate-stage HCC more than the best supportive care [14]. However, TACE is not recommended for patients with a poor liver function, as it may lead to liver failure [15].

Alcohol consumption has been proven to cause cirrhosis and is the main risk factor for developing HCC [3]. Besides, chronic alcohol exposure promotes HCC stemness and metastasis [16]. However, some patients with alcohol addiction fail to quit drinking, despite being diagnosed with HCC. In China, all patients diagnosed with HCC are advised to quit alcohol consumption. However, some patients continue drinking after undergoing liver resection, RFA, or TACE. Therefore, this study explored the influence of continuous alcohol consumption in patients with HCC who underwent TACE using data from two centers.

## 2. Materials and Methods

Patients from the two centers were retrospectively reviewed between January, 2014, and December, 2021. A total of 254 patients who consumed alcohol before TACE were included in the study. Among them, 101 patients continued to consume alcohol and 153 quit drinking (alcohol abstinence is continuous abstinence from alcohol for at least 12 weeks [17]). This study was conducted in accordance with the principles of the Declaration of Helsinki. This study was approved by the ethics committees of the two centers. The requirement for informed consent from the patients was waived by the ethics committees as the study was retrospective.

The inclusion criteria were as follows: (1) patients diagnosed with HCC based on imaging and/or laboratory examination; (2) patients who received TACE; (3) patients consuming alcohol (the amount of alcohol consumption is equivalent to more than 3 alcoholic units (AU)/d or 21 AU/week (with 1 AU containing 12 grams of ethanol) [18]) before receiving TACE; (4) Child-Pugh A or B liver function; (5) Eastern Cooperative Oncology Group (ECOG) performance score of 0 or 1; and (6) platelet count > 60 × 10^9^/L.

**Figure 1:**
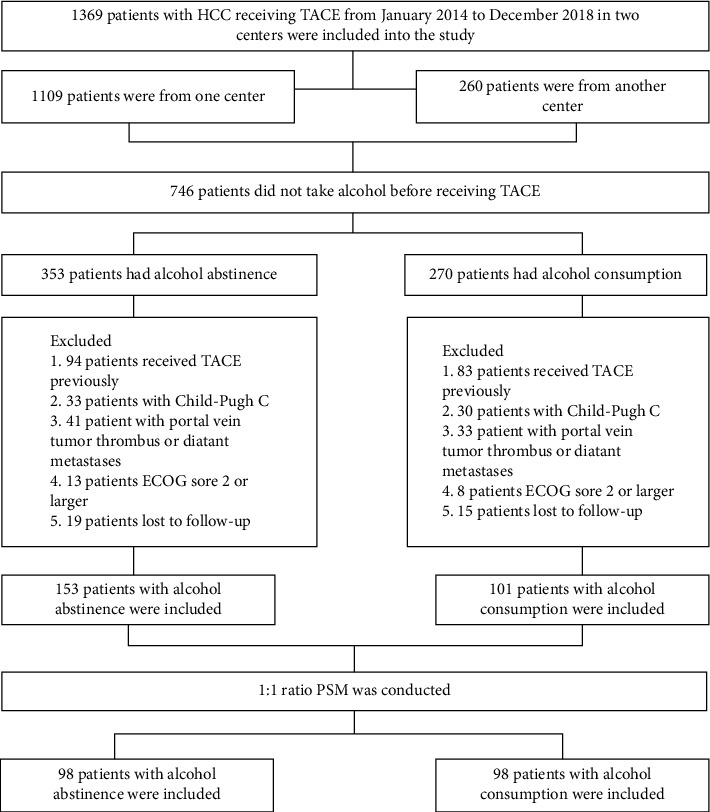
Flowchart of patient selection.

The exclusion criteria were as follows: (1) patients who underwent TACE, RFA, or liver resection before inclusion in the study; (2) patients with portal vein tumor thrombus; (3) patients with diffuse tumors; and (4) patients lost during follow-up (Figure 1).

### 2.1. Transarterial Chemoembolization Procedure

All TACE procedures were conducted by multidisciplinary teams, including specialists with >10 years of experience. The procedure was performed under local anesthesia via the right femoral artery. Under digital subtraction angiography guidance, a super-selective microcatheter was inserted into the feeding artery of the tumor via selective hepatic angiography. A mixture of 5–20 mL of Lipiodol (Lipiodol Ultra Fluid; French: Guerbet) and 10–40 mg of doxorubicin hydrochloride (Hisun Pharmaceutical Co. Ltd., Zhejiang, China) was mixed and injected into the selected artery through the microcatheter; the exact dose depended on each patient's embolization condition. Finally, the feeding arteries were embolized using gelatin sponge particles until complete stasis of the arterial flow.

### 2.2. Study Endpoints

The primary endpoints were overall survival (OS) and progression-free survival (PFS). The secondary point of the study was the objective response rate (ORR) of the patients at 6 months after TACE. OS was defined as the interval from the time of initial TACE to that of the patient's death or the end of the study. PFS was defined as the interval from the time of initial TACE to that of tumor progression, the patient's death, or the end of the study based on the modified Response Evaluation Criteria in Solid Tumors (mRECIST) [19]. ORR was defined as the proportion of patients with a complete response (CR) and partial response (PR).

### 2.3. Evaluation of Adverse Events

Safety was assessed based on adverse events. The adverse events in patients were evaluated and graded based on the National Cancer Institute Common Terminology Criteria for Adverse Events (NCI-CTCAE) version 4.03, and the seriousness of the adverse events was recorded [20].

### 2.4. Follow-Up

All patients included in the study were followed up. The patients underwent imaging (computed tomography (CT) or magnetic resonance imaging (MRI)) and laboratory examinations to evaluate the tumor response and liver function. The interval for each follow-up was 4–6 weeks for three months after the initial TACE and 6–8 weeks thereafter. The imaging (CT or MRI) data of the patients were evaluated by a radiologist (with 15 years of experience) and an interventional radiologist (with 31 years of experience). If the tumor progressed, patients were recommended to undergo another TACE. The study ended in April, 2022.

### 2.5. Statistical Analysis

All statistical analyses were performed using R version 4.1.2 (R core development team, 2010), and the imaging results were plotted using GraphPad Prism 9.3.0. All *P* < 0.05 were considered to be statistically significant. Continuous variables between the two groups were compared using the independent sample *t*-test or Mann–Whitney *U* test. Categorical variables were compared between the two groups using the chi-square test or Fisher's exact test. The survival curves in the two groups were plotted using the Kaplan–Meier method and compared using the log-rank test. The Cox model was used to predict potential factors that may influence outcomes in all patients. Variables with *P* < 0.05 in the univariate regression analysis were included in the multivariate regression analysis.

PSM was performed to reduce selection bias. All baseline characteristics were included in the PSM analysis. A 1 : 1 ratio of the nearest neighbor matching was performed with an optimal caliper of 0.1. After PSM, 98 pairs of patients were matched.

There are several noncancer-related causes, such as trauma and cardiovascular disease, that may lead to death before the onset of cancer-related death; thus, it is necessary to examine the possible influence of competing events on the association between alcohol consumption status and survival using Fine and Gray's sub-distribution hazards regression model. Competing risk analysis using noncancer-related deaths as competing events was performed to evaluate the survival of patients before and after PSM.

In addition, we performed four sets of sensitivity analyses using adjusted multivariate Cox models in the original sample and the population after PSM. Models 1 and 3 were adjusted for age and sex for the OS and PFS, respectively. Considering that some risk factors were identified in the univariate and multivariate regression analyses, models 2 and 4 were adjusted for age, sex, neutrophil-to-lymphocyte ratio (NLR), platelet-to-lymphocyte ratio (PLR), albumin level, bilirubin level, maximum tumor size, TACE session, and ECOG PS.

## 3. Results

### 3.1. Patients

A total of 254 patients who underwent TACE were included in this study. Of which, 101 continued to consume alcohol and 153 had alcohol abstinence. In the alcohol consumption group, a total of 84 (83.2%) patients died and 78 (77.2%) died due to cancer during follow-up. In the alcohol abstinence group, a total of 119 patients (77.8%) died and 107 (69.9%) died due to cancer during follow-up. Two patients died due to liver failure after the second TACE in the alcohol consumption group. Before PSM, the characteristics of the Barcelona Clinic Liver Cancer (BCLC) stage and ECOG PS were unbalanced. After PSM, all baseline characteristics of the two groups were balanced (Table 1).

### 3.2. Survival Outcomes

**Figure 2:**
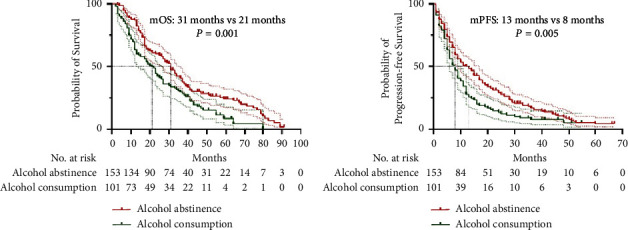
Kaplan–Meier survival curves for patients in both the groups before propensity score matching. (a) Kaplan–Meier curve for overall survival (OS). (b) Kaplan–Meier curve for progression-free survival (PFS).

**Figure 3:**
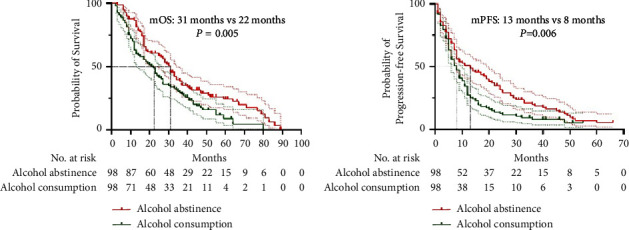
Kaplan–Meier survival curves for patients in both the groups after propensity score matching. (a) Kaplan–Meier curve for overall survival (OS). (b) Kaplan–Meier curve for progression-free survival (PFS).

Before PSM, the mOS in the alcohol abstinence group (31 months, 95% confidence interval (CI): 26.8–35.2 months) was significantly longer than that in the alcohol consumption group (21 months, 95% CI: 15.8–26.2 months; *P*=0.001) (Figure 2). Moreover, before PSM, the mPFS in the alcohol abstinence group (13 months, 95% CI: 9.4–16.6 months) was significantly longer than that in the alcohol consumption group (8 months, 95% CI: 6.1–9.9 months; *P*=0.005) (Figure 2(b)). After PSM, the mOS in the alcohol abstinence group (31 months, 95% CI: 26.8–35.2 months) remained significantly longer than that in the alcohol consumption group (22 months, 95% CI: 16.4–27.6 months; *P*=0.005) (Figure 3). The mPFS in the alcohol abstinence group (13 months, 95% CI: 5.8–20.2 months) also remained significantly longer than that in the alcohol consumption group (8 months, 95% CI: 6.0–10.0 months; *P*=0.006) (Figure 3(b)).

### 3.3. Tumor Response after 6 Months

Before PSM, the CR, PR, stable disease (SD), and progressive disease (PD) in the alcohol consumption group were similar to those in the alcohol abstinence group (all *P* > 0.05). However, the ORR (57.5%, 88/153) in the alcohol abstinence group was higher than that in (42.6, 43/101) alcohol consumption group (*P*=0.003). After PSM, the CR in the alcohol abstinence group (19, 19.4%) was higher than that (9, 9.2%) in the alcohol consumption group (*P*=0.041). The PR, SD, and PD in the alcohol abstinence group were similar to those in the alcohol consumption group (all *P* > 0.05) (Table 2).

### 3.4. Predictors for OS and PFS

Before PSM, the multivariate regression analysis suggested that albumin levels, maximum tumor size (hazard ratio (HR): 0.978, 95% CI: 0.958–0.999; *P*=0.042), maximum tumor size (HR: 1.069, 95% CI: 1.028–1.110; *P*=0.001), TACE session (HR: 0.367, 95% CI: 0.244–0.551; *P* < 0.001), and alcohol consumption (HR: 1.486, 95% CI: 1.074–2.055; *P*=0.016) were independent predictors of OS (Table 3). Furthermore, the multivariate regression analysis showed that maximum tumor size (HR: 1.072, 95% CI: 1.032–1.115; *P* < 0.001), TACE session (HR: 0.439, 95% CI: 0.296–0.652; *P* < 0.001), and alcohol consumption (HR: 1.434, 95% CI: 1.091–1.886; *P*=0.01) were independent predictors of PFS (Table 4).

### 3.5. Competing Risk Analysis and Sensitivity Analysis

After excluding deaths caused by other reasons, the multivariate regression analysis showed that alcohol consumption increased the mortality risk more than alcohol abstinence did before PSM (HR: 1.377, 95% CI: 1.005–1.886; *P*=0.046) (Table 5) and after PSM (HR: 1.505, 95% CI: 1.060–2.134; *P*=0.022) (Table 6).

Further, four sensitive analysis models were constructed. In models 1 and 3, the variables age and sex were adjusted. In model 1, the analysis suggested that alcohol consumption increased the mortality risk (HR: 1.655, 95% CI: 1.255–2.234; *P*=0.001) and tumor progression risk (HR: 1.456, 95% CI: 1.112–1.907; *P*=0.006) more than alcohol abstinence before PSM (Table 7). In model 3, similar results were obtained after PSM. Alcohol consumption increased the mortality risk (HR: 1.572, 95% CI: 1.141–2.166; *P*=0.006) and tumor progression risk (HR: 1.507, 95% CI: 1.111–2.045; *P*=0.008) more than alcohol abstinence (Table 8).

In models 2 and 4, the variables age, sex, NLR, PLR, albumin levels, bilirubin levels, maximum tumor size, TACE session, and ECOG PS were adjusted. In model 2, the analysis suggested that alcohol consumption increased the mortality risk (HR: 1.561, 95% CI: 1.132–2.150; *P*=0.007) and tumor progression risk (HR: 1.416, 95% CI: 1.060–1.891; *P*=0.018) compared with alcohol abstinence before PSM (Table 7). In model 4, after PSM, alcohol consumption increased the mortality risk (HR: 1.594, 95% CI: 1.144–2.223; *P*=0.006) and tumor progression risk (HR: 1.592, 95% CI: 1.159–2.185; *P*=0.004) compared with that by alcohol abstinence (Table 8).

### 3.6. Changes in the Liver Function Three Months after TACE and Adverse Events Analysis before PSM

**Figure 4:**
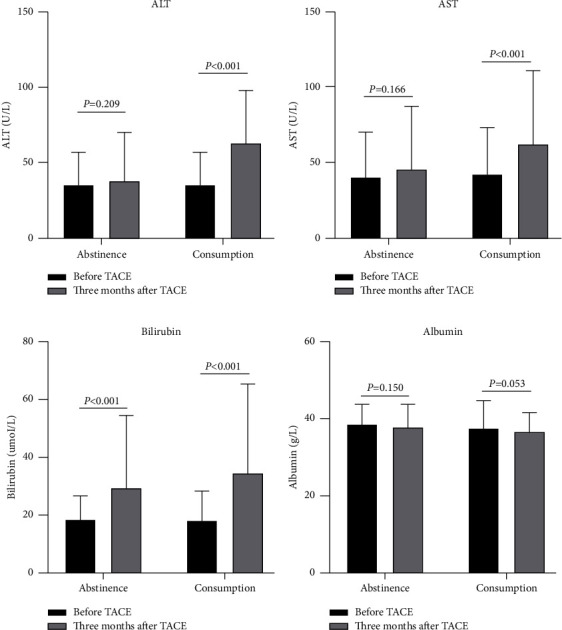
Changes in the liver function indexes before transarterial chemoembolization (TACE) and at three months after TACE before propensity score matching. (a) Change in alanine aminotransferase (ALT). (b) Change in aspartate aminotransferase (AST). (c) Change in bilirubin levels. (d) Change in albumin levels.

In the alcohol consumption group, alanine aminotransferase (ALT), aspartate aminotransferase (AST), and bilirubin levels at three months after TACE were higher than those before TACE. However, in the alcohol abstinence group, only bilirubin levels at three months after TACE were higher than those before TACE (Figure 4).

The adverse event analysis showed that all grades of nausea (47.5% vs. 20.2%; *P* < 0.001) and vomiting (23.8% vs. 13.1%; *P*=0.028) in the alcohol consumption group were higher than those in the alcohol abstinence group. For grade III or IV adverse events, the incidence of nausea (6.9% vs. 1.3%; *P*=0.043) was higher in the alcohol consumption group than that in the alcohol abstinence group (Table 9).

## 4. Discussion

Many studies have focused on the effects of alcohol consumption on the etiology of HCC or liver cirrhosis [21–23]. However, only a few studies have explored the effect of continuous alcohol consumption in patients with HCC who received related treatments. TACE has been widely used to treat HCC. Although previous studies have presented alcohol-related liver disease as an independent factor affecting the survival of patients with HCC who received TACE [24], the effects of continuous alcohol consumption in patients with HCC remain unclear. Thus, this study explored whether continuous alcohol consumption could influence the survival and liver function of patients who underwent TACE.

The main findings of the study were that patients with HCC who received TACE and continued to consume alcohol had poor survival and higher tumor progression risk than patients with alcohol abstinence. Previous retrospective and prospective studies have shown that the mOS in patients with HCC who received TACE ranges between 19.4 and 37 months, and the median time to progression (mTTP) ranges between 3 and 11 months [25–28]. In the current study, 8, 163, and 83 patients with BCLC stage A, stage B, and stage C cancer, respectively, were included. However, all patients with the BCLC stage C cancer had an ECOG PS of 1. Patients with portal vein tumor thrombi or distant metastases were excluded. The results showed that the mOS of patients with alcohol abstinence was 31 months and that of patients consuming alcohol was 21 months. The mOS in patients with alcohol abstinence in this study was higher than that in patients in previous randomized controlled trials (RCT). The mPFS was used to evaluate tumor responses. The difference between PFS and TTP is that the patient's death is censored for TTP. The mPFS in patients with alcohol abstinence in this study was higher than that in previous RCTs. This evidence suggests that alcohol consumption may lead to a poor prognosis in patients with HCC who have undergone TACE. In the Cox model, after excluding other factors that may influence the outcomes, alcohol consumption increased the risk of all-cause mortality and tumor progression compared with alcohol abstinence. To evaluate the robustness of the Cox model used in this study, we analyzed the four sensitive models. The results suggested that alcohol consumption increased the risk of mortality and tumor progression compared with alcohol abstinence. This indicated that patients may benefit from alcohol abstinence.

In this study, 18 patients died of other causes (traffic accidents, cardiovascular disease, etc.). To exclude the influence of death from other causes, a competing risk analysis was conducted. Multivariate regression analysis showed that alcohol consumption continued to increase the mortality risk compared with alcohol abstinence. These results indicate a better prognosis in patients with alcohol abstinence.

HCC usually occurs in people with preexisting hepatic fibrosis. 80–90% of patients with HCC had cirrhosis [3]. In the current study, 65.3% (166/254) patients had cirrhosis, which was lower than previous study reports [3]. The reason might be that only the patients with cirrhosis were recorded, but the patients with mild liver fibrosis were not recorded in the study. Previous studies have shown that alcohol consumption can damage liver function and cause and aggravate cirrhosis [29–31]. Thus, changes in the liver function indices were evaluated. The results showed that patients with alcohol consumption had poor liver function three months after undergoing TACE. However, in the alcohol abstinence group, bilirubin levels were higher before TACE than in the three months after TACE. Albumin levels in both groups failed to increase before TACE and for three months after TACE. This may be because most patients received albumin treatment. The results of this study suggest that alcohol could also damage liver function in patients undergoing TACE. A meta-analysis evaluated the association between alcohol consumption and pain and showed that alcohol consumption could increase chronic pain [32]. However, in the present study, alcohol consumption failed to increase TACE-related abdominal pain risk compared with alcohol abstinence. Alcohol consumption increased all grades of nausea and vomiting risk and grade III or IV nausea risk than that with alcohol abstinence. These results indicate that patients with alcohol abstinence had a better physical status than patients who consumed alcohol after TACE.

This study has some limitations. First, PSM was performed, but some potential selection biases could not be excluded. Second, the sample size of this study was small. Thus, we hope that future RCTs will include large sample sizes to confirm the conclusions of this study.

## 5. Conclusions

Alcohol consumption may lead to a poor prognosis, damage liver function, and increase adverse events compared with alcohol abstinence in patients who undergo TACE. Alcohol abstinence may be needed in patients with HCC who receive TACE.

## Figures and Tables

**Table 1:** Baseline characteristics of patients before and after propensity score matching.

Characteristics	*Before matching*			*After matching*		
	Alcohol use	Alcohol abstinence	*P* value	Alcohol use	Alcohol abstinence	*P* value
Age (years)	55.8 ± 8.8	56.4 ± 10.6	0.640	55.7 ± 8.7	55.8 ± 10.8	0.953
AST	35.2 ± 21.1	35.3 ± 21.8	0.984	41.8 ± 30.5	40.4 ± 29.8	0.735
ALT	52.2 ± 42.4	47.3 ± 34.9	0.562	34.7 ± 20.6	36.5 ± 22.5	0.552
Bilirubin	18.1 ± 10.2	17.6 ± 8.9	0.650	18.4 ± 10.2	16.7 ± 8.7	0.209
Albumin	35.3 ± 21.8	38.9 ± 5.0	0.213	38.0 ± 6.8	38.6 ± 5.1	0.486
NLR	2.5 ± 2.0	3.1 ± 3.0	0.331	2.5 ± 2.1	2.8 ± 2.7	0.496
PLR	106.7 ± 64.8	121.9 ± 78.4	0.108	107.3 ± 65.6	107.7 ± 55.4	0.955
Max tumor size (cm)	7.0 ± 3.8	6.4 ± 3.5	0.156	7.1 ± 3.8	6.5 ± 3.7	0.298

*Gender*			0.618			0.788
Male	92	142		90	91	
Female	9	11		8	7	

*Smoking*			0.534			0.881
Yes	64	91		64	63	
No	37	62		34	35	

*HBV infection*			0.081			0.534
Yes	89	122		86	83	
No	12	31		12	15	

*HCV infection*			1.000			0.718
Yes	4	7		3	5	
No	97	146		95	93	

*Cirrhosis*			0.280			0.771
Yes	62	104		59	57	
No	39	49		39	41	

*Ascites*			0.980			0.809
Yes	10	18		10	9	
No	91	135		88	89	

*TACE session*			0.099			0.841
Once	17	15		15	14	
Multiple times	84	138		83	84	

*Tumor number*			0.512			0.436
1	35	47		32	27	
≥2	66	106		66	71	

*BCLC stage*			<0.001			0.479
A	3	5		3	4	
B	56	107		56	63	
C	42	41		39	31	

*AFP level*			0.358			0.881
<200	63	104		63	64	
≥200	38	49		35	34	

*Child-Pugh*			0.083			>0.999
A	74	126		73	73	
B	27	27		25	25	

*ECOG score*			0.014			0.275
0	59	112		59	67	
1	42	41		39	32	

**Table 2:** Tumor response at 6 months after undergoing transarterial chemoembolization (TACE) between the two groups before and after propensity score matching.

Tumor response	*Before matching*			*After matching*		
	Alcohol use (N/%)	Alcohol abstinence (N/%)	*P* value	Alcohol use (N/%)	Alcohol abstinence (N/%)	*P* value
Complete response	9 (8.9)	26 (17.0)	0.067	9 (9.2)	19 (19.4)	0.041
Partial response	34 (36.7)	62 (40.5)	0.270	33 (33.7)	32 (32.7)	0.879
Stable disease	40 (39.6)	49 (32.0)	0.215	39 (39.8)	32 (32.6)	0.298
Progressive disease	18 (17.8)	16 (10.5)	0.092	17 (17.3)	15 (15.3)	0.699
Objective rate	42.6%	57.5%	0.003	42.9%	52%	0.198

**Table 3:** Univariate and multivariate regression analyses for overall survival before propensity score matching.

Characteristics	*Univariable analysis*		*Multivariable analysis*	
	HR (95% CI)	*P* value	HR (95% CI)	*P* value
Age (years)	0.995 (0.982, 1.009)	0.491		
AST	0.999 (0.992, 1.007)	0.864		
ALT	1.005 (0.999, 1.010)	0.090		
Bilirubin	1.013 (0.997, 1.029)	0.124		
Albumin	0.966 (0.945, 0.988)	0.003	0.978 (0.958, 0.999)	0.042
NLR	1.063 (1.012, 1.116)	0.015	1.048 (0.993, 1.107)	0.091
PLR	1.001 (0.999, 1.003)	0.190		
Maximal tumor size (cm)	1.063 (1.025, 1.102)	<0.001	1.069 (1.028, 1.110)	0.001

*Gender*				
Male	Ref	0.870		
Female	0.959 (0.582, 1.580)			

*Smoking*				
Yes	Ref	0.481		
No	1.107 (0.835, 1.446)			

*HBV infection*				
Yes	Ref	0.412		
No	0.861 (0.602, 1.231)			

*HCV infection*				
Yes	Ref	0.498		
No	0.876 (0.689, 1.212)			

*Cirrhosis*				
Yes	Ref	0.200		
No	0.823 (0.611, 1.109)			

*Ascites*				
Yes	Ref	0.161		
No	0.732 (0.474, 1.132)			

*TACE session*				
Once	Ref	<0.001	Ref	<0.001
Multiple times	0.325 (0.219, 0.481)		0.367 (0.244, 0.551)	

*Tumor number*				
1	Ref	0.341		
≥2	1.159 (0.855, 1.572)			

*BCLC stage*				
A	Ref			
B	1.526 (0.704, 3.304)	0.284		
C	2.150 (0.973, 4.749)	0.058		

*AFP level*				
<200	Ref	0.245		
≥200	1.191 (0.887, 1.600)			

*Child-Pugh*				
A	Ref	0.731		
B	1.059 (0.764, 1.468)			

*ECOG score*				
0	Ref	0.014	Ref	0.284
1	1.444 (1.077, 1.936)		1.185 (0.869, 1.615)	

*Alcohol*				
Abstinence	Ref	0.002	Ref	0.016
Consumption	1.618 (1.194, 2.194)		1.486 (1.074, 2.055)	

**Table 4:** Univariate and multivariate regression analyses for progression-free survival before propensity score matching.

Characteristics	*Univariable analysis*		*Multivariable analysis*	
	HR (95% CI)	*P* value	HR (95% CI)	*P* value
Age (years)	0.994 (0.981, 1.008)	0.402		
AST	1.002 (0.997, 1.007)	0.410		
ALT	1.000 (0.993, 1.007)	0.926		
Bilirubin	1.012 (0.998, 1.026)	0.103		
Albumin	0.981 (0.959, 1.003)	0.093		
NLR	1.026 (0.980, 1.074)	0.276		
PLR	1.000 (0.999, 1.002)	0.757		
Maximal tumor size (cm)	1.068 (1.028, 1.109)	0.001	1.072 (1.032, 1.115)	<0.001

*Gender*				
Male	Ref	0.789		
Female	1.067 (0.665, 1.711)			

*Smoking*				
Yes	Ref	0.357		
No	1.133 (0.869, 1.478)			

*HBV infection*				
Yes	Ref	0.803		
No	1.044 (0.746, 1.460)			

*HCV infection*				
Yes	Ref	0.872		
No	0.989 (0.929, 1.102)			

*Cirrhosis*				
Yes	Ref	0.076		
No	0.777 (0.589, 1.026)			

*Ascites*				
Yes	Ref	0.109		
No	0.719 (0.481, 1.076)			

*TACE session*				
Once	Ref	<0.001	Ref	<0.001
Multiple times	0.413 (0.279, 0.610)		0.439 (0.296, 0.652)	

*Tumor number*				
1	Ref	0.135		
≥2	1.243 (0.935, 1.653)			

*BCLC stage*				
A	Ref			
B	1.280 (0.626, 2.616)	0.499		
C	1.503 (0.723, 3.126)	0.275		

*AFP level*				
<200	Ref	0.729		
≥200	1.050 (0.795, 1.388)			

*Child-Pugh*				
A	Ref	0.523		
B	1.104 (0.815, 1.495)			

*ECOG score*				
0	Ref	0.215		
1	1.192 (0.903, 1.573)			

*Alcohol*				
Abstinence	Ref	0.007	Ref	0.010
Consumption	1.452 (1.109, 1.900)		1.434 (1.091, 1.886)	

**Table 5:** Competing risk analysis for the survival of patients before propensity score matching.

Characteristics	*Competing risk analysis for survival*			
	*Univariable analysis*		*Multivariable analysis*	
	HR (95% CI)	*P* value	HR (95% CI)	*P* value
Age (years)	0.993 (0.980, 1.006)	0.266		
AST	1.004 (0.999, 1.009)	0.108		
ALT	1.000 (0.993, 1.008)	0.931		
Bilirubin	1.021 (1.004, 1.038)	0.014	1.027 (1.010, 1.043)	0.002
Albumin	0.990 (0.963, 1.016)	0.454		
NLR	1.046 (0.996, 1.098)	0.072		
PLR	1.000 (0.998, 1.003)	0.643		
Maximal tumor size (cm)	1.064 (1.024, 1.106)	0.001	1.072 (1.027, 1.119)	0.001

*Gender*				
Male	Ref	0.866		
Female	1.034 (0.700, 1.528)			

*Smoking*				
Yes	Ref	0.176		
No	1.210 (0.917, 1.595)			

*HBV infection*				
Yes	Ref	0.590		
No	0.916 (0.667, 1.259)			

*HCV infection*				
Yes	Ref	0.602		
No	0.899 (0.625, 1.199)			

*Cirrhosis*				
Yes	Ref	0.116		
No	0.784 (0.579, 1.062)			

*Ascites*				
Yes	Ref	0.630		
No	0.880 (0.523, 1.479)			

*TACE session*				
Once	Ref	0.016	Ref	0.059
Multiple times	0.496 (0.280, 0.878)		0.542 (0.287, 1.025)	

*Tumor number*				
1	Ref			
≥2	1.076 (0.793, 1.459)	0.638		

*BCLC stage*				
A	Ref			
B	1.113 (0.649, 1.909)	0.697		
C	1.575 (0.884, 2.802)	0.122		

*AFP level*				
<200	Ref	0.879		
≥200	1.025 (0.743, 1.415)			

*Child-Pugh*				
A	Ref	0.980		
B	1.004 (0.699, 1.442)			

*ECOG score*				
0	Ref	0.027	Ref	0.143
1	1.424 (1.041, 1.948)		1.289 (0.917, 1.813)	

*Alcohol*				
Abstinence	Ref	0.004	Ref	0.046
Consumption	1.529 (1.143, 2.046)		1.377 (1.005, 1.886)	

**Table 6:** Competing risk analysis for the survival of patients after propensity score matching.

Characteristics	*Competing risk analysis for survival*			
	*Univariable analysis*		*Multivariable analysis*	
	HR (95% CI)	*P* value	HR (95% CI)	*P* value
Age (years)	0.991 (0.977, 1.005)	0.196		
AST	1.002 (0.993, 1.010)	0.689		
ALT	0.996 (0.987, 1.006)	0.432		
Bilirubin	1.024 (1.005, 1.043)	0.011	1.025 (1.006, 1.045)	0.011
Albumin	0.999 (0.972, 1.027)	0.928		
NLR	1.068 (1.013, 1.127)	0.016	1.004 (0.918, 1.098)	0.927
PLR	1.004 (1.001, 1.006)	0.007	1.002 (0.999, 1.005)	0.096
Maximal tumor size (cm)	1.067 (1.025, 1.111)	0.002	1.065 (1.018, 1.114)	0.006

*Gender*				
Male	Ref	0.908		
Female	1.027 (0.649, 1.625)			

*Smoking*				
Yes	Ref	0.162		
No	1.255 (0.913, 1.727)			

*HBV infection*				
Yes	Ref	0.872		
No	0.967 (0.647, 1.445)			

*HCV infection*				
Yes	Ref	0.768		
No	0.944 (0.879, 1.267)			

*Cirrhosis*				
Yes	Ref	0.255		
No	0.826 (0.595, 1.147)			

*Ascites*				
Yes	Ref	0.731		
No	1.117 (0.595, 2.095)			

*TACE session*				
Once	Ref	0.030	Ref	0.061
Multiple times	0.513 (0.281, 0.937)		0.527 (0.270, 1.029)	

*Tumor number*				
1	Ref	0.224		
≥2	1.254 (0.871, 1.801)			

*BCLC stage*				
A	Ref			
B	1.208 (0.653, 2.237)	0.547		
C	1.520 (0.797, 2.901)	0.204		

*AFP level*				
<200	Ref	0.777		
≥200	1.053 (0.734, 1.511)			

*Child-Pugh*				
A	Ref	0.763		
B	1.063 (0.714, 1.582)			

*ECOG score*				
0	Ref	0.167	Ref	
1	1.274 (0.903, 1.798)			

*Alcohol*				
Abstinence	Ref	0.005	Ref	0.022
Consumption	1.596 (1.151, 2.185)		1.505 (1.060, 2.134)	

**Table 7:** Sensitivity analysis before propensity score matching. Model 1: adjusted for age and sex. Model 2: adjusted for age, sex, neutrophil-to-lymphocyte ratio (NLR), platelet-to-lymphocyte ratio (PLR), albumin levels, bilirubin levels, maximum tumor size, transarterial chemoembolization (TACE) session, and Eastern Cooperative Oncology Group (ECOG) performance score.

	Alcohol status	*Model one*		*Model two*	
		HR (95% CI)	*P* value	HR (95% CI)	*P* value
OS	Abstinence	Ref	0.001	Ref	0.007
	Consumption	1.655 (1.225, 2.234)		1.561 (1.132, 2.150)	

PFS	Abstinence	Ref	0.006	Ref	0.018
	Consumption	1.456 (1.112, 1.907)		1.416 (1.060, 1.891)	

**Table 8:** Sensitivity analysis after propensity score matching. Model 3: adjusted for age and sex. Model 4: adjusted for age, sex, neutrophil-to-lymphocyte ratio (NLR), platelet-to-lymphocyte ratio (PLR), albumin levels, bilirubin levels, maximum tumor size, transarterial chemoembolization (TACE) session, and Eastern Cooperative Oncology Group (ECOG) performance score.

	Alcohol status	*Model three*		*Model four*	
		HR (95% CI)	*P* value	HR (95% CI)	*P* value
OS	Abstinence	Ref	0.006	Ref	0.006
	Consumption	1.572 (1.141, 2.166)		1.594 (1.144, 2.223)	

PFS	Abstinence	Ref	0.008	Ref	0.004
	Consumption	1.507 (1.111, 2.045)		1.592 (1.159, 2.185)	

**Table 9:** Adverse event analysis for patients consuming alcohol and those with alcohol abstinence after undergoing transarterial chemoembolization (TACE).

Adverse events	*All grades*			*III or IV grades*		
	Alcohol use (N/%)	Alcohol abstinence (N/%)	*P* value	Alcohol use (N/%)	Alcohol abstinence (N/%)	*P* value
Fever	26 (25.7)	39 (25.4)	0.964	2 (2.0)	3 (2.0)	>0.999
Abdominal pain	35 (34.7)	37 (24.2)	0.070	1 (1.0)	2 (1.3)	>0.999
Nausea	48 (47.5)	33 (20.2)	<0.001	7 (6.9)	2 (1.3)	0.043
Vomit	24 (23.8)	20 (13.1)	0.028	1 (1.0)	1 (0.7)	>0.999
Poor appetite	58 (57.4)	71 (46.4)	0.086	2 (2.0)	1 (0.7)	0.564
Cholecystitis	4 (4.0)	7 (4.6)	>0.999	1 (1.0)	1 (0.7)	>0.999
Liver abscess	2 (2.0)	3 (2.0)	>0.999	0 (0)	0 (0)	—

## Data Availability

The data used in the study are available from the corresponding author on reasonable request.
